# Personality and intelligence: persistence, not self-directedness, cooperativeness or self-transcendence, is related to twins’ cognitive abilities

**DOI:** 10.7717/peerj.1195

**Published:** 2015-08-20

**Authors:** Fariba Mousavi, Sandor Rozsa, Thomas Nilsson, Trevor Archer, Henrik Anckarsäter, Danilo Garcia

**Affiliations:** 1 Network for Empowerment and Well-Being, Sweden; 2 Institute of Neuroscience and Physiology, Centre for Ethics, Law and Mental Health, University of Gothenburg, Gothenburg, Sweden; 3 Department of Psychology, University of Gothenburg, Gothenburg, Sweden; 4 Center for Well-Being, Department of Psychiatry, Washington University School of Medicine in St. Louis, St. Louis, MO, USA

**Keywords:** Cognitive ability, Cooperativeness, Intelligence, Self-directedness, Temperament and character inventory, Twins

## Abstract

**Background.** A person-centered approach focusing on the interaction of an individual’s temperament-character-life events is essential in the path of individuals’ well-being. In this context, three character traits, Self-directedness (e.g., self-acceptance, self-control, goal-directed behavior), Cooperativeness (e.g., social affiliation, social tolerance, empathy and helpfulness) and Self-transcendence (e.g., spiritual acceptance, transpersonal identification), measured using Cloninger’s model of personality are suggested to help the individual to regulate and resolve the conflicts derived from her/his temperament combinations as a reaction to life events. However, if character is related to the individual’s cognitive ability, then this association might limit any intervention that focuses on character development. We used data from the Child and Adolescent Twin Study in Sweden (CATSS) to investigate the relationship between personality and cognitive ability.

**Method.** The sample consisted of 370 15-year-old twins (159 girls/211 boys), 192 of whom screen-positive with various types of mental health problems. We used the Temperament and Character Inventory to measure personality and the Wechsler Intelligence Scales for Children (WISC-IV) to measure intelligence. The relationship was investigated using correlation analyses using random-selected twins from each dyad and separately for monozygotic and dizygotic twins. Additional analyses investigated the genetic and environmental effects on personality and cognitive ability in this specific sample.

**Results.** There were no significant correlations between the WISC-IV indices and any of the character traits (i.e., Self-directedness, Cooperativeness, and Self-transcendence). Persistence was significantly related, if weak, to four WISC-IV indices: Verbal Comprehension, Perceptual Reasoning, Working Memory, and the Full WISC-IV Scale. Post-hoc cross-twin/cross-trait analyses showed that the Persistence-cognitive ability correlation might depend on common genetic effects. The WISC-IV indices showed a relatively large genetic influence, while earlier findings about the etiology of temperament and character traits using the whole CATSS sample were replicated in this sub-sample of twins.

**Conclusions.** The results indicate that what individuals make of themselves intentionally (i.e., their character) was not associated to intelligence. Persistence, a temperament dimension that measures heritable individual differences in eagerness of effort, ambition, perfectionism, and resistance to discouragement despite frustration and fatigue, was weakly linked to intelligence. Suggesting that, at least during adolescence, interventions targeting character development are not limited by the individual’s intelligence.

Cloninger’s psychobiological model of personality (Cloninger, Svrakic & Przybeck, 1993; Cloninger et al., 1994) comprises four temperament and three character dimensions. The four temperament dimensions are: Harm Avoidance (i.e., being worry, fearful, easily getting tired, shy, doubtful and pessimistic), Novelty Seeking (i.e., the tendency to respond and seek new stimuli, rapid loss of temper and impulsive decision making), Reward Dependence (i.e., the tendency to respond to signals of reward, social approval, and sentiment), and Persistence (i.e., the propensity to persevere in behaviors despite frustration and fatigue). Peculiarly, Persistence was a later addition to the model and recognized as a fourth separately inherited dimension of personality (Gillespie et al., 2003) related to an anatomical brain circuit that includes parts of the medial network of orbital and adjacent medial prefrontal cortex, which are involved in the modulation of social behaviour and the control of mood and motivational drive (Gusnard et al., 2003). High persistence is associated with resilience and positive emotionality (Garcia, 2011), being industrious and persevering (Gusnard et al., 2003). The three character dimensions are: Self-directedness (i.e., self-esteem and self-reliance, self-acceptance, and the tendency of being responsible, reliable, goal-oriented, constructive and having well-integrated good habits), Cooperativeness (i.e., the individual’s acceptance of other people, supportive and respect for the preferences and needs of others, and the tendency of being empathetic, tolerant, and compassionate), and Self-transcendence (i.e., the individual’s sense of being part of nature and the universe, and the tendency to experience higher levels of insight meditation). That is, while the temperament dimensions refer to automatic reactions to stimuli, the character dimensions refer to what the individual make of her/himself intentionally (cf. Cloninger, 2004).

In this context, different temperament combinations are predictors of specific disorders but not of who has a disorder or not (see Cloninger, 2004). For example, while individuals with AD/HD are characterized by high scores in Novelty Seeking, not all individuals with this temperament combination end up with a AD/HD diagnosis and the negative outcomes of such behaviour. In contrast, the character traits (i.e., Self-directedness, Cooperativeness, and Self-transcendence) are correlated to higher levels of well-being (Cloninger, 2004; Cloninger, Svrakic & Svrakic, 1997; Cloninger & Zohar, 2011) and lower levels of dysfunction and suffering associated to neuropsychiatric symptoms among children and adolescents (Garcia, Anckarsäter & Lundström, 2013). In other words, personality is a dynamic adaptive system in which the individual’s personality, temperament and character, interact within the person and with life events and circumstances to maintain homeostasis but also well-being (see Cloninger, 2004). Indeed, hampered development of character has been related to increased risk for a broad range of mental health problems, including personality disorders (Svrakic et al., 1993) and to both AD/HD and autism spectrum disorders in children (Kerekes et al., 2013), adolescents (Garcia, Anckarsäter & Lundström, 2013), and adults (Anckarsater et al., 2006). Thus, a mature character development indicates a sense of unity of the self (unity with the self, others and some universal transcendental sense of the being) that helps the individual to regulate conflicts that might derive from different temperament configurations (Cloninger, 2004).

Character, in contrast to temperament, has been found in recent prospective studies to increase with age (Josefsson et al., 2013). These character traits are positively associated to psychophysiological coherence, a state of calm alertness that occurs naturally with sustained positive emotions and can be induced by slow, deep breathing, relaxing, and sleeping; which increases efferent parasympathetic activity (Zohar, Cloninger & McCraty, 2013). This supports the notion that a more mature and developed character is associated with more peripheral nervous system inhibitory activity, perhaps as a form of mature emotional regulation that arises from an outlook of unity, connectedness, and harmony with the self, others and the world around (Cloninger, 2004; Cloninger, Zohar & Cloninger, 2010; Cloninger, 2013). This sense of unity allows a person to function efficiently in a state of calm awareness without unhealthy psychophysiological arousal, defensiveness, and negative emotions in the face of the challenges of daily life (Bradley et al., 2010; McCraty et al., 2009; Cloninger, 2013). In other words, suggesting at least phenotypical (if not causal) relations among personality, heart rate variability, and health (i.e., the absence of ill-being and the presence of well-being).

Interventions that improve character (referred to as Well-Being Therapy/Coaching) lead to alleviation of destructive behaviour patterns and mental disorders, and to increased positive emotions, life satisfaction, sense of meaning, and well-being as a whole (e.g., Albieri et al., 2009; Cloninger, 2006; Cloninger, 2013; Fava, 1999; Fava et al., 1998a; Fava et al., 1998b; Fava et al., 2005; Moeenizadeh & Salagame, 2010). In turn, neurobiological evidence shows that individuals who tend to frequently experience positive affect have lower cortisol levels (Cohen et al., 2003) and a strengthened immune response (Rosenkranz et al., 2003). Nevertheless, a well-developed character could also be thought to result from general cognitive maturity and ability, thus, suggesting that positive character development is epiphenomenal rather than an independent psychological feature. Consequentially, interventions aimed to increase character might therefore be constrained by intelligence.

The concept of general intelligence, or “g”, refers to the common ability underlying performance on all cognitive tests. This ability could be described in terms of a number of specific, but still quite general cognitive abilities under the notion of “g”. The most widely used distinction is between fluid and crystallized intelligence (Kan et al., 2013). Fluid intelligence describes abilities that are innate and not necessarily dependent on previous training or experience while crystallized intelligence describes abilities that depend on acquired knowledge, training, or experience (Kan et al., 2013). Fluid and crystallized intelligence are inter-correlated, and most intelligence tests attempt to measure both. For example, the Wechsler Intelligence Scale for Children (WISC-IV; Wechsler, 2003) measures fluid intelligence using perceptual tasks and crystallized intelligence using verbal tasks.

In a study using adults with schizophrenia and their non-psychotic siblings (Smith et al., 2008), researchers found that while temperament and character were not associated to cognitive abilities in persons with schizophrenia, both Self-directedness and Cooperativeness were correlated to crystallized intelligence in unaffected siblings as measured by a battery of neuropsychological tests composed of subtests of Wechsler Memory Scale and the California Verbal Learning Test (see Harms et al., 2008; Delawalla et al., 2006). Smith and colleagues (2008) concluded that the positive associations between intelligence and both Self-directedness and Cooperativeness among non-psychotic siblings suggest that a more maturely developed character (i.e., autonomic, responsible, tolerant, helpful, and empathic behavior) together with higher levels of crystallized intelligence and other neurocognitive functioning may enhance resilience or protection against schizophrenia. Studies among children with externalizing behaviour problems (e.g., Copeland et al., 2004) have found that parent-reported Self-directedness was positively related to intelligence as measured by shorter versions of Weschler scales measuring both fluid and crystallized intelligence. In addition, researchers have shown that the temperament dimension of Persistence, which is associated to parts of the medial network of the orbital and medial prefrontal cortex, is important in reward-related activities, including the expectation and detection of reward (Gusnard et al., 2003) and a significant predictor of academic success and related to intelligence as measured by Cattell’s Reasoning Scale (Moreira et al., 2012; Poropat, 2009). These findings suggest that personality, as measured in Cloninger’s model, has specific associations to intelligence.

However, the number of studies investigating the relationship between personality traits, measured by the Cloninger model, and intelligence are few (for a review see Moreira et al., 2012). In addition, studies using the Big Five Model of personality (Costa & McCrae, 1992) show that certain personality traits, independently from intelligence, predict academic achievement (for a Meta-Analysis see Poropat, 2009). Specifically, high levels of Conscientiousness (i.e., an individual defined as effective, organized, ambitious, hardworking, and thoughtful), high levels of Agreeableness (i.e., an individual friendly, empathic, gentle, modest, tolerant, forgiving and peaceful), and high levels of Openness (i.e., an individual defined as imaginative, idealistic, inventive, artistic, affectionate, and unconventional) were independently from intelligence associated to academic performance (Poropat, 2009; see also Laidra, Pullman & Allik, 2007). These Big Five personality traits are related to the character dimensions Self-directedness, Self-transcendence, and Cooperativeness of Cloninger’s model of personality. De Fruyt and colleagues (2005) for example, found positive associations among psychiatric patients between Conscientiousness-Self-directedness, Agreeableness-Cooperativeness, and Openness-Self-transcendence. These specific findings were replicated in a more recent study among Swedish high school pupils (Garcia, 2012). Nevertheless, one significant advantage of Cloninger’s model is that it comprises personality traits that, if maladaptive, are central to clinical interest (Widiger & Lowe, 2007), such as Self-directedness, Cooperativeness, and Self-transcendence.

In sum, among individuals with mental disorders, the results are mixed: character traits are in some studies not related to intelligence (e.g., Smith et al., 2008) and in other studies significantly related (Copeland et al., 2004). The present study uses data from a clinical sub-study of the Child and Adolescent Twin Study in Sweden (CATSS; Anckarsäter et al., 2011), referred to as “Developmental Outcomes in a Genetic twin Study in Sweden” (DOGSS, Anckarsäter et al., 2011) to test the relationship between personality as measured by Cloninger’s model and cognitive ability as measured by Wechsler intelligence scales.

## Method

### Ethical statement

The study has ethical approval from the Karolinska Institute Ethical Review Board: Dnr: 03-6722010/1356/31/1. The Participants are protected by informed consent process—they are informed of what is being collected and were repeatedly given the option to withdraw their consent and discontinue their participation. All adolescents in the study had written consent from parents, caretakers, or guardians to participate in the study.

### Participants and procedure

The present study is based on a sample of 452 twins (DOGSS), aged 15 years. A total of 370 twins out of the 452 included in the study completed both the personality and the intelligence tests (159 girls, 211 boys), with a total of 163 monozygotic and 207 dizygotic twins (192 screen-positive children, 138 screen-negative co-twins, and 40 randomly chosen controls matched for sex and age). A detailed description of the whole population (*N* = 452) is presented in Larson and colleagues’ study (2013). The personality data for the whole CATSS sample, including the DOGSS, has been analysed in an earlier study by Garcia and colleagues (2013).

The recruitment procedure is thoroughly described by Anckarsäter and colleagues (2011). Briefly, same-sex twins pairs born between January 1st, 1993—the 31st of December 1995, in whom at least one of the twins had screened positive for autism spectrum disorders, ADHD, learning disabilities, developmental coordination disorder and/or a related behavioural disorder, such as obsessive compulsive disorder, oppositional defiant disorder, conduct disorder, and/or eating disorder, in telephone interviews at age 9/12, were invited to participate. In addition, a number of randomly selected population controls were included. The twins and their parents were assessed by licensed psychologists who were blind to all previous information and to the results of the examination of the co-twin. The clinical assessment included, among other things, the WISC-IV. In connection to the clinical assessment, questionnaires including the Temperament and Character Inventory, were sent to the twins in advance to fill out, and deliver at the clinical assessment.

### Measures

#### Personality

The Temperament and Character Inventory (TCI; Cloninger, Svrakic & Przybeck, 1993; Cloninger et al., 1994) comprises 238 items with binary answers (*true* = 1, false = 0). Examples of questions from the four temperament dimensions are: Harm Avoidance, “I often feel tense and worried in unfamiliar situations, even when others feel there is little to worry about” (Cronbach alpha =.84); Novelty Seeking, “I often try new things just for fun or thrills, even if most people think it is a waste of time” (Cronbach alpha =.77); Reward Dependence, “I like to discuss my experiences and feelings openly with friends instead of keeping them to myself” (Cronbach alpha =.68); and Persistence, “I often push myself to the point of exhaustion or try to do more than I really can” (Cronbach alpha =.61). Examples of questions from the three character dimensions: Self-directedness, “In most situations my natural responses are based on good habits that I have developed” (Cronbach alpha =.82); Cooperativeness, “I often consider another person’s feelings as much as my own” (Cronbach alpha =.83); and Self-transcendence, “I sometimes feel so connected to nature that everything seems to be part of one living organism” (Cronbach alpha =.83).

### Cognitive ability

The fourth revised version of Wechsler Intelligence Scale for Children (WISC-IV; Wechsler, 2003) was used here. The scale is intended for assessment of cognitive ability among children and adolescents (6–16 years). The structure of the scale is based on a division of indexes: (1) Verbal Comprehension, tasks that evaluate skills and understanding on verbal information, thinking and reasoning with words, and expressing thoughts as words; (2) Perceptual Reasoning, tasks that evaluate skills in solving nonverbal problems, sometimes using eye-hand coordination, and working quickly and efficiently with visual information; (3) Working Memory, tasks that measure skills in attention, concentration, and mental reasoning, which is closely related to learning and achievement; (4) Processing Speed, tasks that measure skills in speed of mental problem-solving, attention, and eye-hand coordination. A Full Scale IQ score, a combination of the scores of all four indexes, measures overall thinking and reasoning skills. Each index is composed of sub-scales. The Verbal Comprehension index consists of: Similarities, Vocabulary and Comprehension; the Perceptual Reasoning index consists of: Block Pattern, Photo Categories, Matrices (Picture Completion); the Working Memory index consists of: Digit Repetition, Letter-Number Series, (Arithmetic); and the Processing Speed Index of: Coding and Symbol Search.

#### Zygosity

Zygosity was determined by a validated algorithm based on five questions on twin similarity, derived from 571 pairs of twins with known zygosity. Only twins with more than a 95% probability of being correctly classified, compared to DNA-testing, were assigned zygosity by this method (Hannelius et al., 2007).

### Statistical analysis

Preliminary analyses were performed for the TCI and WISC-IV variables to investigate violation of the assumptions of normality, outliers, linearity and homoscedasticity. The test of Normality of the Kolmogorov–Smirnov statistic was non-significant, which indicates no violation of the assumption of normality. Moreover the shape of the distribution in the histograms was judged as normally distributed and also was supported by an inspection of the normal probability plots (Normal Q-Q Plot). Inspection of descriptive statistics showed that the original mean and 5% trimmed mean values were very similar, thus, there were no extreme scores (i.e., outliers) that had a strong influence on the mean. Scatterplots of the variables showed roughly straight lines, not curves; hence the relationship between the two variables was judged as linear. Finally, homoscedasticity was checked by the variability in scores. The variance of the dependent variable was the same for all the data. All main statistical tests (two-tailed) were conducted using parametric methods in SPSS version 20.

In order to avoid confounding effects due to twin pair intra-dependence in the whole sample including both twins of each dyad, the main correlation analyses were performed using twins #2 from each pair (*n* = 184; 81 girls and 103 boys; 80 monozygotic and 104 dizygotic) to investigate the relationship between personality trait (as measured by the Temperament and Character Inventory) and cognitive ability (as measured by the WISC-IV indices). Moreover the same analyses were also re-conducted on the whole twins samples 1 and 2 with and without mental health problems (*n* = 138 screen-negative co-twins and 40 randomly chosen controls and *n* = 192 screen-positive children). Post hoc Power analysis showed a *δ* between .56 and .73 for the correlations conducted in this study.

Significant associations between personality and intelligence scales were further examined using cross-twin/cross-trait analyses to address if these relationships depended on genetic effects. In addition, the data was analyzed using twin methodology, which is basically a comparison of monozygotic twins, who are genetically identical, and dizygotic twins who, on average, share 50% of their segregating alleles. As a first step, intraclass correlation coefficients for the seven dimensions in the TCI were calculated separately for monozygotic twins and same-sex dizygotic twins. As a second step, we performed univariate genetic analyses, using a model-fitting approach with structural equation-modeling techniques, using Mplus. By comparing the difference of intraclass correlation coefficients between monozygotic and dizygotic twins it is possible to disentangle the genetic and environmental contribution to a trait. The genetic and environmental contributions are partitioned into three variance components: genetic factors (A), shared environmental factors that make the twins similar (C) and non-shared, unique environmental factors that make the twins dissimilar (E).

## Results

### Correlation analysis between personality and cognitive ability for screen-negative and positive twins

As a first part of the analysis, we investigated the correlations between personality and intelligence for screen-negative and screen-positive children (cf. Smith et al., 2008, who found such a difference between healthy individuals and individuals with schizophrenia). The results for screen-negative group show a weak positive correlation between Persistence and Verbal Comprehension: *r* = .18, *p* < 0.05 (*r*^2^ = .03), Working Memory: *r* = .16, *p* < 0.05 (*r*^2^ = .03) and the Full Scale IQ: *r* = .15, *p* < .05 (*r*^2^ = .02) and even a weak positive correlation was found between Self-transcendence and Perceptual Reasoning: *r* = .19, *p* < .05 (*r*^2^ = .04), Cooperativeness: *r* = .16, *p* < 0.05 (*r*^2^ = .03) and Working Memory, Cooperativeness: *r* = .17, *p* < 0.05 (*r*^2^ = .03) and Processing Speed. For screen-positive groups the correlation show a weak relationship between Persistence and Verbal Comprehension: *r* = .24, *p* < 0.01 (*r*^2^ = .06), Perceptual Reasoning: *r* = .18, *p* < .05 (*r*^2^ = .03), Working Memory: *r* = .16, *p* < 0.05 (*r*^2^ = .03) and the Full Scale IQ: *r* = .23, *p* < .01 (*r*^2^ = .05) (See Table 1).

**Table 1:** Correlation matrix of the seven TCI dimensions and WISC- IV indices for screen-negative and positive twins (whole sample, that is, twins #1 and #2). 138 screen-negative co-twins and 40 randomly chosen controls (*N* = 178) (blue fields) and screen-positive children *N* = 192 (white fields).

		1	2	3	4	5	6	7	8	9	10	11	12
Temperament	Novelty Seeking (1)		−.15^*^	−.02	−.31^**^	−.27^**^	.03	−.32^**^	−.084	−.05	−.09	−.04	−.03
	Harm Avoidance (2)	−.16^*^		.10	−.22^**^	−.08	−.04	−.44^**^	.00	.06	.07	.11	.10
	Reward Dependence (3)	.02	.12		.17^*^	.60^**^	.26^**^	.19^*^	−.06	−.01	.09	.08	.04
	**Persistence (4)**	−.40^**^	−.13	.04		.28^**^	.19^**^	.27^**^	.18^*^	.10	.16^*^	.08	.15^*^
Character	Cooperativeness (5)	−.15^*^	−.12	.43^**^	.26^**^		.28^**^	.45^**^	.04	.07	.16^*^	.17^*^	.10
	Self-transcendence (6)	.00	.09	.10	.08	.10		−.09	−.10	−.19^*^	−.02	−.09	−.11
	Self-directedness (7)	−.29^**^	−.38^**^	.07	.30^**^	.46^**^	−.25^**^		.00	.03	.04	.06	−.02
Cognitive ability	Verbal Comprehension Index (8)	−.07	.03	−.00	.24^**^	.11	−.08	.02		.49^**^	.47^**^	.32^**^	.76^**^
	Perceptual Reasoning Index (9)	−.03	.00	−.03	.18^*^	.04	−.08	.04	.67^**^		.40^**^	.58^**^	.73^**^
	Working Memory Index (10)	−.07	−.05	−.06	.16^*^	.01	−.07	.02	.58^**^	.55^**^		.35^**^	.65^**^
	Processing Speed Index (11)	−.05	.01	.07	.05	.09	−.06	−.04	.42^**^	.54^**^	.44^**^		.64^**^
	Full Scale IQ (12)	−.07	.02	.00	.23^**^	.09	−.08	.016	.90^**^	.85^**^	.75^**^	.67^**^	

**Notes.**

* Correlation is significant at the 0.05 level (2-tailed).

** Correlation is significant at the 0.01 level (2-tailed).

Additional analyses showed no statistically significant difference in the strength of the correlation between Persistence and Verbal Comprehension (*z_obs_* = .60), Persistence and Working Memory (*z_obs_* = 0) and Persistence and Full Scale IQ (*z_obs_* = .79) for screen-negative and positive twins. Hence, all separate analysis using twins #2 (or twins #1) were conducted using a mixed sample (i.e., both screen-positive and screen-negative twins).

#### Correlation analysis between personality and cognitive ability for twin #2

Correlation matrices are given in Table 2. Overall the character traits did not show any significant correlations with the intelligence scales, and, among the temperament traits, there were weak, albeit statistically significant positive correlations between Persistence and Verbal Comprehension: *r* = .29, *p* < .01 (*r*^2^ = .08), Perceptual Reasoning: *r* = .22, *p* < .01 (*r*^2^ = .05), Working Memory: *r* = .19, *p* < .01 (*r*^2^ = .04), and the Full Scale IQ: *r* = .26, *p* < .01 (*r*^2^ = .07). In other words, higher levels of Persistence were associated with higher levels of Verbal Comprehension, Perceptual Reasoning, Working Memory and Full Scale IQ (details given in Table 2).

**Table 2:** Correlation matrix, mean and standard deviation of the seven personality dimensions and all four WISC-IV indices and IQ score using twins #2.

		1	2	3	4	5	6	7	8	9	10	11	12	*M*	*SD*	*N*
Temperament	Novelty Seeking (1)		−.16^*^	−.03	−.37^**^	−.15^*^	.02	−.29^**^	−.07	−.14	−.05	−.01	−.08	22.33	5.98	184
	Harm Avoidance (2)			.12	−.13	−.12	−.02	−.49^**^	−.03	.05	−.03	.08	.05	15.89	6.55	184
	Reward Dependence (3)				.25^**^	.55^**^	.26^**^	.12	−.06	−.05	.09	.09	.02	13.78	3.87	184
	**Persistence (4)**					.34^**^	.21^**^	.26^**^	**.29** ^**^	**.22** ^**^	**.19** ^**^	.11	**.26** ^**^	4.18	2.08	184
Character	Cooperativeness (5)						.19^*^	.44^**^	.05	.02	.11	.13	.08	29.78	6.11	184
	Self-transcendence (6)							−.19^**^	−.08	−.11	.01	−.01	−.05	11.49	5.79	184
	Self-directedness (7)								.01	.02	.01	−.06	−.02	27.64	6.86	184
Cognitive ability	Verbal comprehension Index (8)									.66^**^	.59^**^	.46^**^	.89^**^	97.50	21.93	184
	Perceptual Reasoning Index (9)										.54^**^	.64^**^	.86^**^	101.20	15.67	184
	Working Memory Index (10)											.46^**^	.75^**^	93.91	15.43	184
	Processing Speed Index (11)												.73^**^	93.45	16.43	184
	Full Scale IQ (12)													96.19	17.99	181

**Notes.**

* Correlation is significant at the 0.05 level (2-tailed).

** Correlation is significant at the 0.01 level (2-tailed).

### Correlation analysis between personality and cognitive ability for twin #1

The same analyses were conducted also for twins #1. In contrast to the same analyses conducted with twin #2, the correlation analyses using twin #1 showed a small positive correlation between Self-transcendence and Processing Speed: *r* = .15, *p* < .05 (*r*^2^ = .02) and small negative correlations between Self-directedness and the following cognitive ability measures: Verbal Comprehension: *r* = − .15, *p* < .05 (*r*^2^ = .02), Perceptual Reasoning: *r* = − .21, *p* < .01 (*r*^2^ = .04), Working Memory: *r* = − .18, *p* < .05 (*r*^2^ = .03), Processing Speed: *r* = − .22, *p* < .01 (*r*^2^ = .05) and Full Scale IQ: *r* = − .20, *p* < .01 (*r*^2^ = .04). Persistence, however, was not significantly related to any WISC-IV index or the Full Scale IQ among the twin #1 sample (See Table 3).

**Table 3:** Correlation matrix, mean and standard deviation of the seven personality dimensions and all four WISC-IV indices and IQ score using twins #1.

		1	2	3	4	5	6	7	8	9	10	11	12	*M*	*SD*	*N*
Temperament	Novelty Seeking (1)		−.16^*^	.03	−.35^**^	−.32^**^	−.26^**^	.01	−.06	.09	−.08	−.06	.01	22.67	5.94	186
	Harm Avoidance (2)			.09	−.23^**^	−.35^**^	−.09	.09	.05	−.02	.00	−.01	.04	15.70	6.27	186
	Reward Dependence (3)				−.04	.16^*^	.49^**^	.12	−.01	.00	−.08	.07	.02	13.53	3.90	186
	Persistence (4)					.32^**^	.19^**^	.06	.12	.06	.11	.01	.11	3.88	1.92	186
Character	Cooperativeness (5)						.47^**^	−.16^*^	.03	.08	.08	.11	.03	27.58	6.93	186
	Self-transcendence (6)							.19^*^	.12	.11	.05	.15^*^	.13	28.47	6.72	186
	Self-directedness (7)								−.15^*^	−.21^**^	−.18^*^	−.22^**^	−.20^**^	11.79	5.57	186
Cognitive ability	Verbal Comprehension Index (8)									.56^**^	.52^**^	.36^**^	.80^**^	95.18	23.18	182
	Perceptual Reasoning Index (9)										.50^**^	.52^**^	.77^**^	10.33	15.17	181
	Working Memory Index (10)											.40^**^	.71^**^	92.92	13.72	182
	Processing Speed Index (11)												.63^**^	92.38	14.11	181
	Full Scale IQ (12)													94.11	17.90	181

**Notes.**

* Correlation is significant at the 0.05 level (2-tailed).

** Correlation is significant at the 0.01 level (2-tailed).

### Correlation analysis between personality and cognitive ability for twin #2 divided by zygocity

The correlations were also re-calculated dividing the sample of twins #2 by zygocity (80 monozygotic and 104 dizygotic). The relationship between WISC-IV indices and personality showed small positive correlations in the monozygotic group between the Persistence and Verbal Comprehension: *r* = .27, *p* < .05 (*r*^2^ = .07), Perceptual Reasoning: *r* = .28, *p* < .05 (*r*^2^ = .08) and the Full Scale IQ: *r* = .26, *p* < .05 (*r*^2^ = .07). In the dizygotic group, there were relationships between the Persistence and Verbal Comprehension: *r* = .31, *p* < .01 (*r*^2^ = .10), Working Memory *r* = .21, *p* < .05 (*r*^2^ = .04) and the Full Scale IQ: *r* = .25, *p* < .01 (*r*^2^ = .06). See Table 4 for the details. Additional analyses showed no statistically significant difference in the strength of the correlation between Persistence and Verbal Comprehension (*z_obs_* = 0.63) and Persistence and Full Scale IQ (*z_obs_* = 0.07) for monozygotic and dizygotic twins.

**Table 4:** Correlation matrix of the seven TCI dimensions and WISC-IV indices for monozygotic and dizygotic (twins #2). Monozygotic *N* = 80 (blue fields), dizygotic *N* = 104 (white fields).

		1	2	3	4	5	6	7	8	9	10	11	12
Temperament	Novelty Seeking (1)		−.34^**^	−.09	−.47^**^	−.17	−.09	−.06	−.07	−.19	.01	−.00	−.09
	Harm avoidance (2)	−.07		.09	.06	−.29^**^	.02	−.04	.05	.17	−.04	.07	.13
	Reward dependence (3)	.02	.15		.20	.19	.66^**^	.31^**^	−.10	−.02	.07	.06	−.03
	**Persistence (4)**	−.30^**^	−.27^**^	.29^**^		.21	.23^*^	.11	**.27** ^*^	.28^*^	.19	.14	**.26** ^*^
Character	Cooperativeness (5)	−.36^**^	−.60^**^	.07	.32^**^		.44^**^	−.23^*^	.01	.01	−.04	−.06	−.04
	Self-transcendence (6)	−.18	−.22^*^	.46^**^	.44^**^	.45^**^		.26^*^	.06	.04	.15	.16	.11
Self-directedness (7)	.07	−.01	.22^*^	.29^**^	−.15	.13		−.04	−.07	.04	.01	.01
Cognitive ability	Verbal Comprehension Index (8)	−.07	−.09	−.04	**.31** ^**^	.02	.04	−.11		.65^**^	.59^**^	.52^**^	.90^**^
	Perceptual Reasoning Index (9)	−.11	−.04	−.08	.16	.03	.00	−.14	.67^**^		.51^**^	.63^**^	.85^**^
	Working Memory Index (10)	−.08	−.03	.10	**.21** ^*^	.04	.08	−.02	.59^**^	.55^**^		.52^**^	.73^**^
	Processing Speed Index (11)	−.02	.09	.11	.09	−.04	.11	−.03	.42^**^	.66^**^	.43^**^		.76^**^
	Full Scale IQ (12)	−.08	−.01	.05	**.25** ^**^	.00	.06	−.08	.88^**^	.87^**^	.76^**^	.70^**^	

**Notes.**

* Correlation is significant at the 0.05 level (2-tailed).

** Correlation is significant at the 0.01 level (2-tailed).

To assess whether the relation between Persistence and these specific intelligence scales could be referred to shared genetic effects influencing both features, post-hoc correlations were also calculated cross-twin/cross-trait separately for monozygotic and same-sex dizygotic pairs—the Persistence score of twin #1 was correlated to Verbal Comprehension and Full Scale IQ in twin #2. For monozygotic twins, Persistence was significantly related to both Verbal Comprehension (*r* = .33, *p* < .01) and Full Scale IQ (*r* = .25, *p* < .05). For dizygotics, Persistence was not significantly related to either Verbal Comprehension (*r* = .10, *p* = .344) or Full Scale IQ (*r* = .10, *p* = .335). A Fisher’s test showed statistically significant difference in the strength of the correlation between Persistence and Verbal Comprehension (*z_obs_* = 2.3) but not statistically significant difference between Persistence and Full Scale IQ (*z_obs_* = 1.4). In other words, the correlation between Persistence and Verbal Comprehension was stronger for monozygotic pairs than for dizygotic pairs. This may indicate that the phenotypic correlation may be primarily referred to common genetic effects that influence both Persistence and Verbal Comprehension, but not Persistence and Full Scale IQ.

The correlations were also conducted among twin #2 (monozygotic and dizygotic) by gender, Table 5 shows the differences between the outcome variable groups regarding TCI dimensions and WISC- IV indices. Additional analyses showed that there is not a statistically significant difference in the strength of the correlation between Persistence and Verbal Comprehension for males and females (*z_obs_* = − 1.20). Persistence dose not explain significantly more of the variance in Verbal Comprehension for females than for males.

**Table 5:** Correlation matrix of the seven TCI dimensions and WISC-IV indices for gender monozygotic and dizygotic, twins #2. Male *N* = 103 (blue fields), female *N* = 81 (white fields).

		1	2	3	4	5	6	7	8	9	10	11	12
Temperament	Novelty Seeking (1)		−.30^**^	−0.08	−.42^**^	−0.16	−0.14	−0.06	−0.10	−0.15	−0.12	−0.05	−0.14
	Harm avoidance (2)	−0.03		0.08	−0.11	−.39^**^	−0.05	−0.16	−0.12	−0.03	−0.08	−0.00	−0.04
	Reward dependence (3)	−0.06	0.07		.25^*^	0.13	.49^**^	0.18	−0.12	−.24^*^	0.14	−0.17	−0.10
	**Persistence (4)**	−.31^**^	−0.18	.24^*^		.25^**^	.33^**^	.29^**^	.22^*^	0.19	0.17	−0.03	0.18
Character	Cooperativeness (5)	−.42^**^	−.57^**^	0.2	.29^**^		.44^**^	−0.08	−0.02	−0.04	−0.00	−0.13	−0.06
	Self-transcendence (6)	−0.21	−.27^*^	.58^**^	.34^**^	.49^**^		.20^*^	−0.00	−0.13	0.07	−0.04	−0.02
	Self-directedness (7)	0.08	0.09	.25^*^	0.08	−.27^*^	0.11		−0.16	−.23^*^	0.01	−0.12	−0.15
Cognitive ability	Verbal Comprehension Index (8)	−0.01	0.08	0.04	.39^**^	0.02	0.13	0.05		.67^**^	.57^**^	.45^**^	.89**
	Perceptual Reasoning index (9)	−0.13	0.13	0.13	.25^*^	0.09	0.19	0.03	.67^**^		.54^**^	.59^**^	.86^**^
	Working Memory Index (10)	0.05	0.02	0.04	.23^*^	0.02	0.16	−0.01	.62^**^	.53^**^		.49^**^	.76**
	Processing Speed Index (11)	−0.07	0.06	0.09	.25^*^	0.08	.22^*^	−0.05	.59^**^	.77^**^	.48^**^		.70^**^
	Full Scale IQ (12)	−0.03	0.13	0.10	.34^**^	0.03	0.19	0.04	.91^**^	.86^**^	.74^**^	.81^**^	

**Notes.**

* Correlation is significant at the 0.05 level (2-tailed).

** Correlation is significant at the 0.01 level (2-tailed).

### ACE model

Table 6 presents results for fitting separate univariate ACE models to each personality dimension and WISC-IV indices. The point estimate for the proportion of variance accounted for by additive genetic effects (A) was the largest for Perceptual Reasoning. In contrast to any of the temperament dimensions, all character dimensions (i.e., Self-directedness, Cooperativeness, and Self-transcendence) together with Verbal Comprehension, Working Memory, Processing Speed, and Full Scale IQ showed a shared environmental effect (C). The unique environmental estimates (E) for all variables in the study were over 0.45 and statistically significant.

**Table 6:** Univariate ACE model results.

		*A* ^2^	*C* ^2^	*E* ^2^	*χ* ^2^	df	p	CFI	TLI	RMSEA
Temperament	Novelty Seeking	0.53	0.00	0.47	8.314	6	.216	.906	.969	.069
	Harm avoidance	0.52	0.00	0.48	6.950	6	.325	.959	.986	.044
	Reward dependence	0.59	0.00	0.41	1.693	6	.945	1.000	1.000	.021
	Persistence	0.36	0.00	0.64	11.758	6	.067	.586	.862	.108
Character	Cooperativeness	0.34	0.28	0.38	5.839	6	.441	1.000	1.000	.000
	Self-transcendence	0.31	0.24	0.45	2.146	6	.905	1.000	1.000	.000
	Self-directedness	0.39	0.24	0.37	5.815	6	.444	1.000	1.000	.000
Cognitive ability	Verbal Comprehension Index	0.54	0.22	0.25	2.561	6	.861	1.000	1.000	.000
	Perceptual Reasoning Index	0.65	0.00	0.35	3.743	6	.711	1.000	1.000	.000
	Working Memory Index	0.54	0.11	0.35	7.648	6	.265	.967	.989	.058
	Processing Speed Index	0.28	0.23	0.48	10.444	6	.107	.877	.959	.095
	Full Scale IQ	0.59	0.08	0.33	9.079	6	.169	.945	.982	.079

**Notes.**

*A*^2^ proportion of variance attributed to additive genetic influences *C*^2^ proportion of variance for shared environment *E*^2^ proportion of variance for nonshared environment and error

## Discussion

The aim of the present study was to investigate the relationship between personality traits (as measured by the Temperament and Character Inventory) and cognitive ability (as measured by the WISC-IV indices). The results from the current study show that there was a lack of significant relationships between character (i.e., Self-directedness, Cooperativeness, and Self-treanscendence) and the cognitive ability scales. There were, however, some discrepancies between the results for twin #1 and #2 and also between genders. For example, in contrast to results for twin #2, for twin #1 we found small relationships between cognitive ability and both Self-transcendence and Self-directedness.

Our findings are in line to earlier adult studies among non-psychotic siblings of schizophrenics (Smith et al., 2008) that show positive relations between these character traits and crystallised intelligence. The correlations between personality and cognitive ability in the present study, however, were lower than those found by Smith and colleagues and, in most of the cases, under what is recommended as “practically” significant effect for social science data (see Ferguson, 2009). Nevertheless, at least in comparison to findings among adults, it is important to point out that intelligence test scores reflect how far the individual’s performance deviates from the average performance of others who are the same age (Stoslopf, 2009). As character, in contrast to temperament, increases with age (Josefsson et al., 2013), so does crystallized intelligence. This is partially supported by the results of the ACE model; in which shared environmental effects were found for all character dimensions and Verbal Comprehension (WISC-IV’s measure of crystallized intelligence). That being said, our findings are also slightly different from those among children with externalized behavioural problems (Copeland et al., 2004), which showed parent-reported Self-directedness as positively associated to intelligence. Although it is plausible to suggest that this might be due to the different methods used in the present study (e.g., self-report instead of parent-report for the personality measures, full-version of the WISC-IV instead of WISC-III sub-sets, clinical rather that self-administration), we suggest that our findings should be replicated before arriving to a definitive conclusion regarding the relationship between character and intelligence.

Furthermore, the temperament trait Persistence was significantly related to some of the cognitive ability scales: Verbal Comprehension, Perceptual Reasoning, Working Memory and Full Scale IQ. This finding is in line with previous research showing that high Persistence, which is involved in the modulation of social behaviour and the control of mood and motivational drive (Gusnard et al., 2003) and effort (Cloninger et al., 1994), defines individuals who are industrious and persevering. Our results suggest that a persistent individual has cognitive abilities for thinking and reasoning with words and expressing thoughts as words (i.e., Verbal Comprehension). Moreover, high persistence is associated to the ability to solve nonverbal problems and working quickly and efficiently with visual information (i.e., Perceptual Reasoning). Finally, a highly persistent individual has also skills in attention, concentration, and mental reasoning (i.e., Working Memory). Indeed, Moreira and colleagues (2012) found that Persistence was a predictor of academic achievement, as measured by grade point average over four trimesters in Portuguese and Philosophy, among adolescents (12–18 years of age). Additionally, Persistence is related to activity in the prefrontal cortex and parietal cortex, which also support working memory (DeYoung, Peterson & Higgins, 2005; DeYoung & Gray, 2009) and distributes a common substrate for both intelligence and intellectual engagement (Shamosh et al., 2008). The cross-twin/cross-trait analyses suggested that the observed positive relationships between Persistence and intelligence (i.e., Verbal Comprehension) might depend on common genetic effects—genes giving rise to both Persistence and intelligence, to one of the phenotypes that leads to the other, or to features assessed both as Persistence and intelligence. Furthermore people with higher persistence might have worked harder to solve the items in intelligence tests. Persistence is, for instance coupled to the brain’s noradrenergic system and explains maintenance of behavior (Cloninger et al., 1994). Adolescents high in Persistence are described as hardworking, and stable despite frustration and fatigue. They are also expected to increase their efforts in response to anticipated reward (Garcia, Kerekes & Archer, 2012). In other words, frustration and fatigue may be perceived as a personal challenge, they do not give up easily and are probably willing to make major sacrifices to be a success (e.g., good grades) (Garcia, Kerekes & Archer, 2012). In this context, Fleischhauer and colleagues (2010) found evidence that individuals with higher need for cognition (a trait that overlaps with persistence) did not necessarily answer more reasoning items but answer the items they worked on more accurately than did individuals low in need for cognition.

### Limitations, inquiries for further research, and last remarks

One limitation in the present study is the methods of measurement that were used—personality being self-reported, while intelligence was assessed by clinically administered WISC-IV tests (Sternberg, 1985). Nevertheless, the Temperament and Character Inventory is a reliable instrument to measure personality (Cloninger, 2004). But one potential limitation was that in the version used in the present study, Persistence is measured using only eight items, which might explain the low reliability of this temperament dimension in the present study (Cronbach alpha =.61). Thus, replication of the results presented here are necessary using new and revised versions of the TCI (see http://anthropedia.org/tci/ for the latest versions of the Temperament and Character Inventory).

Another important point is that the two sub-samples (i.e., twins #1 and twins #2) differ in some respects. This was indicated by the fact that the correlations across the character traits are quite different (e.g., Cooperativeness-Self-directedness *r* = − .16 for twins #1 and .44 for twins #2). These specific differences might suggest some selecting bias when twins were assigned their number, bias that cannot be accounted for in this study and might have lead to the divergent findings. For instance, most of the twins #1 are the first born of the dyad, thus, it does not seem to be any systematical randomization. Hence, future longitudinal studies need to replicate our results to assess generalizability.

Moreover, although we used ACE-models to discern genetic and environmental influences, it is important to bear in mind that, the sample consists of same sex monozygotic and dizygotic twins, one or both of whom were screen-positive for one up till seven neurodevelopmental disorders with substantial overlap among them. In addition, the group includes control pairs where none of the twins screened positive for any disorder. In other words, the sample was recruited from the extreme end of a distribution of neurodevelopmental disorders, therefore mainly including pairs that are discordant for up to seven types of neurodevelopmental disorders and numerous of different constellations of these disorders. Thus, due to this uneven sampling, the distribution of the “scales” (i.e., the variance and, importantly, covariance between twins in a pair) in the sample might not be representative of the cohort from which it is drawn. Therefore any conclusion from the ACE-model should be interpreted with caution and replicated among healthy adolescent twin populations.

The results presented here are an addition to the literature showing that character traits are not associated to specific cognitive abilities or intelligence per se (e.g., Moreira et al., 2012). With regard to Persistence, the findings suggest that this temperamental trait is associated to cognitive abilities (i.e., Verbal Comprehension, Perceptual Reasoning, Working Memory) and IQ in general, thus contributing to academic achievement related to grades in language and abstract reasoning from other studies (e.g., Moreira et al., 2012). Nevertheless, the cross-sectional data and a non-significant association between intelligence and personality cannot answer the question of whether interventions targeting character are constrained by intelligence. For instance, intelligence might moderate the effect of the intervention outcome although it’s not related to character. Moreover, the link between character and intelligence might develop after adolescence when the genetic influence of personality and intelligence increases (which might also explain the inconsistency to the results of Smith et al., 2008). That being said, at least during adolescence, interventions targeting character development are not limited by the individual’s intelligence and character seems to be influenced by the shared environment.

*“We must remember that intelligence is not enough.*

*Intelligence plus character – that is the goal of true education.”*

*Martin Luther King Jr.*

## Additional Information and Declarations

### Data Availability

The following information was supplied regarding the deposition of related data: The raw data is only available upon request to the Swedish Twin Registry, lead reseacher Paul Lichtenstein: http://ki.se/en/research/contact-the-swedish-twin-registry.
